# Molecular mechanisms underlying floral trait formation in *Phalaenopsis* orchids

**DOI:** 10.1093/hr/uhaf340

**Published:** 2025-12-10

**Authors:** Fei Wang, Xinyi Zuo, Angel Wingho Sze, Zhimei Li, Tao Xie, Hongyan Shan, Rui Zhang, Ruidong Jia, Hongzhi Kong, Peipei Wang

**Affiliations:** College of Horticulture, Northwest A&F University, Yangling, Shaanxi 712100, China; Traditional Chinese Medicine & Floriculture Research Center, Kunpeng Institute of Modern Agriculture at Foshan, Foshan, Guangdong 528200, China; College of Horticulture, Northwest A&F University, Yangling, Shaanxi 712100, China; Traditional Chinese Medicine & Floriculture Research Center, Kunpeng Institute of Modern Agriculture at Foshan, Foshan, Guangdong 528200, China; Traditional Chinese Medicine & Floriculture Research Center, Kunpeng Institute of Modern Agriculture at Foshan, Foshan, Guangdong 528200, China; Foshan Timely Biotechnology Limited, Foshan, Guangdong 528244, China; Traditional Chinese Medicine & Floriculture Research Center, Kunpeng Institute of Modern Agriculture at Foshan, Foshan, Guangdong 528200, China; School of Agricultural and Biological Engineering, Foshan University, Foshan, Guangdong 528225, China; State Key Laboratory of Systematic and Evolutionary Botany, CAS Center for Excellence in Molecular Plant Sciences, Institute of Botany, Chinese Academy of Sciences, Beijing 100093, China; College of Horticulture, Northwest A&F University, Yangling, Shaanxi 712100, China; State Key Laboratory of Vegetable Bio Breeding, Key Laboratory of Biology and Genetic Improvement of Flower Crops (North China), Ministry of Agriculture and Rural Affairs, Institute of Vegetables and Flowers, Chinese Academy of Agricultural Sciences, Beijing 100081, China; State Key Laboratory of Systematic and Evolutionary Botany, CAS Center for Excellence in Molecular Plant Sciences, Institute of Botany, Chinese Academy of Sciences, Beijing 100093, China; Traditional Chinese Medicine & Floriculture Research Center, Kunpeng Institute of Modern Agriculture at Foshan, Foshan, Guangdong 528200, China; Guangdong Huabo Ecological Industry Co., Ltd., Foshan, Guangdong 528241, China

## Abstract

*Phalaenopsis* orchids are one of the most important ornamental crops, prized for their beautiful flowers and long flowering phase. Hundreds of commercially available cultivars display a remarkable range of variation in key horticultural traits, including inflorescence type, floral size, and color patterning. While most current cultivars have been developed through cross-breeding or mutation breeding, genetic homogenization has become a growing concern. This is largely due to extensive hybridization among existing cultivars, which are predominantly derived from a limited number of parental species. Additionally, trait linkage in *Phal.* can hinder the integration of desirable characteristics in progeny. Therefore, there is an urgent need to decipher the genetic programs governing key horticultural traits to facilitate both conventional and molecular breeding. Despite significant research efforts, progress has been hampered by several resource limitations. These include a scarcity of high-quality genome assemblies, the lack of stable genetic transformation systems, and insufficient materials for molecular biology studies—a challenge exacerbated by the plant’s relatively long life cycle. Consequently, the molecular mechanisms underlying the formation and diversity of most important horticultural traits in *Phal.* orchids remain largely unexplored. This review summarizes recent research advances, with a primary focus on the key floral traits in *Phal.* orchids, including inflorescence type, flowering time, floral organ organization, color patterning, size, longevity, scent, organ shape, cuticle production, and wax biosynthesis. Furthermore, we offer perspectives on future research directions aimed at elucidating the genetic basis for the remarkable diversity of these traits and advancing molecular breeding in *Phal.* orchids.

## Introduction

As one of the most important ornamental crops with significant ornamental and economic value, *Phalaenopsis* orchids exhibit remarkable diversity in floral traits, such as inflorescence structure and type, flowering time and phase, size, symmetry, color patterning (e.g. stripe, spot), organ shape and structure, scent, among others. While there are approximately 75 wild *Phal.* species—primarily distributed across tropical and subtropical regions of Asia and Oceania [1], the commercial market offers hundreds to thousands of cultivars, largely developed through cross breeding or mutation breeding. Notably, certain phenotypic lineages can be traced to specific ancestral species: white large-flowered cultivars mainly descend from *Phalaenopsis amabilis* or *P. aphrodite*; red large-flowered types largely originate from *P. sanderiana*, *P. schilleriana*, and *P. stuartiana*; striped and spotted patterns are primarily derived from *P. lindenii* and *P. stuartiana*, respectively; scented cultivars are mainly progenies of species in the subgenus *Polychilos*; and the petal-like lips characteristic of the ‘Bigfoot’ series (known as ‘Big tongue’ in Mainland China) track back to *P.* World Class ‘Bigfoot’ [2]. Due to the limited genetic pool and extensive hybridization among existing cultivars by breeders and enthusiasts, it has become increasingly challenging to develop novel variants through conventional crossing, and the issue of homogenization becomes increasingly severe. There is thus an urgent need to integrate new technologies—such as genomics-assisted breeding and genome editing—into *Phal.* breeding programs. The effective application of these approaches, however, relies on a deeper understanding of the diversity and genetic mechanisms underlying key horticultural traits.

**Figure 1:**
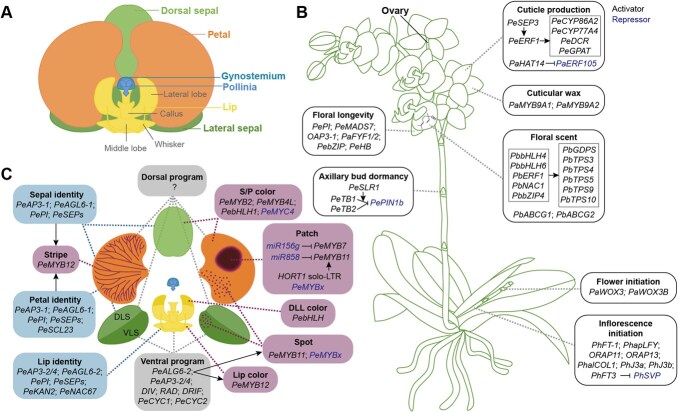
Summary on current knowledge of floral trait formation in *Phalaenopsis* orchids. (A) A *Phal.* orchid flower, with one middle (dorsal) and two lateral (ventral) sepals, two lateral petals, one lip, one gynostemium containing two pollinia, and ovules embedded in the ovary (B). The lip of a typical *Phal.* cultivar contains two lateral lobes, one middle lobe, one thick peltate callus, and two whiskers at the anterior end of the middle lobe. (B) A *Phal.* orchid plant showing current molecular knowledge about inflorescence initiation (flowering time regulation), floral longevity, axillary bud dormancy, flower number determination (flower initiation), floral scent formation, cuticle production and cuticular wax biosynthesis on the perianth. The diagram on the petal depicts the chemical structure of linalool, one of the predominant volatiles in most fragrant *Phal*. orchids. (C) Known genes involved in floral organ identity determination (blue boxes), floral symmetry specification (grey boxes), and color patterning (purple boxes). *Pe*: *P. equestris*; *Pa* and *Phap*: *P. aphrodite* subsp. *formosana*; *Pb*: *P. bellina*; *Ph*: *P.* hybrids; *O* and *OR*: orchids. DLS and VLS: dorsal and ventral region of lateral sepals, respectively; DLL: dorsal region of lateral lobe of the lip. *AP3*: *APETALA3*; *AGL6*: *AGAMOUS-LIKE 6*; *PI*: *PISTILLATA*; *SEP*: *SEPALLATA*; *miR*: *microRNA*; LTR: long terminal repeats; *HORT1*: *Harlequin Orchid RetroTransposon 1*; *SCL*: *STEM CELL LEUKEMIA*; *KAN*: *KANADI*; *NAC*: *NAM, ATAF1/2, and CUC2*; *DIV*: *DIVARICATA*; *RAD*: *RADIALIS*; *DRIF*: *DIV-and-RAD-interacting-factor*; *CYC*: *CYCLOYDEA*; *bHLH*: *basic helix–loop–helix*; *WOX*: *WUSCHEL-related homeobox*; *FYF*: *FOREVER YOUNG FLOWER*; *bZIP*: *basic leucine zipper*; *HB*: *homeobox*; *SLR1*: *Slender Rice 1*; *TB1/2*: *TEOSINTE BRANCHED 1/2*; *ERF*: *ethylene responsive factor*; *HAT*: *HOMEOBOX FROM ARABIDOPSIS THALIANA*; *CYP*: *cytochrome P450*; *DCR*: *defective in cuticular ridges*; *GPAT*: *glycerol-3-phosphate acyltransferase*; *GDPS*: *GERANYL DIPHOSPHATE SYNTHASE*; *TPS*: *terpene synthase*; *ABCG*: *ABC* subfamily *G* gene; *FT*: *FLOWERING LOCUS T*; *LFY*: *LEAFY*; *AP1*: *APETALA1*; *COL*: *CONSTANS*; J3a: J-domain protein 3a; *SVP*: *SHORT VEGETATIVE PHASE*. Information of these genes are listed in Table 1. Arrows: activation; lines with blunt ends: repression.

Although progress has been made in deciphering the genetic basis of some traits in *Phal.*, the molecular mechanisms controlling many important characteristics—such as the vernalization requirement in most cultivars, the sub-rotund petal shape—remain elusive. Crucially, the genetic origins of the striking diversity among these traits are still largely unexplored. In this review, we summarize the recent advances in understanding the genetic regulation of floral traits in *Phal.* orchids (Fig. 1, Table 1). By systematically examining the trait diversity, we identify key research gaps that merit further investigation. Finally, we offer perspectives on future directions for both fundamental research and practical breeding applications in *Phal.* orchids.

**Table 1:** Gene information of *Phal.* orchids mentioned in this study.

**Floral trait involved**	**Gene name**	**Gene ID**	**Database**	**Publication**	**Note**	**ID in GCF_001263595.1**
Flowering time	*PhFT-1*	Not available		Zhou *et al*. [3]	Sequence available in the paper	XP_020599121.1
	*PhapLFY*	KP893636	GenBank	Jang [4]		XP_020598185.1
	*ORAP11*	DQ104328	GenBank	Chen *et al*. [5]		XP_020572933.1
	*ORAP13*	DQ104327	GenBank	Chen *et al*. [5]		XP_020572932.1
	*PhalCOL1*	ACY95395	GenBank	Ke *et al*. [6]		XP_020571252.1
	*PaFVE*	KM496331	GenBank	Koh *et al*. [7]		XP_020577282.1
	*PhJ3a*	Peq002892	OrchidBase 3.0	Jiang *et al*. [8]	Updated to OrchidBase 6.0	XP_020600122.1
	*PhJ3b*	Peq018449	OrchidBase 3.0	Jiang *et al*. [8]	Updated to OrchidBase 6.0	XP_020579216.1
	*PhFT3*	Peq017805	OrchidBase 3.0	Jiang *et al*. [8]	Updated to OrchidBase 6.0	XP_020595880.1
	*PhSVP*	MG560826	GenBank	Jiang *et al*. [8]		XP_020575639.1
	*PaFT*	Not available		Semiarti *et al*. [9]		
	*POH1*	Not available		Semiarti *et al*. [9]		
Floral organ identity	*PeMADS2*	AY378149	GenBank	Tsai *et al*. [10]	*PeAP3-1*	XP_020586439.1
	*PeMADS3*	AY378150	GenBank	Tsai *et al*. [10]	*PeAP3-2*	XP_020571650.1
	*PeMADS4*	AY378147	GenBank	Tsai *et al*. [10]	*PeAP3-4*	XP_020588460.1
	*PeMADS5*	AY378148	GenBank	Tsai *et al*. [10]	*PeAP3-3*	XP_020580539.1
	*PeSEP1*	KF673857	GenBank	Pan *et al*. [11]		XP_020599713.1
	*PeSEP2*	KF673858	GenBank	Pan *et al*. [11]		XP_020591078.1
	*PeSEP3*	KF673859	GenBank	Pan *et al*. [11]		XP_020592894.1
	*PeSEP4*	KF673860	GenBank	Pan *et al*. [11]		XP_020572941.1
	*PhaMADS7*	Not available		Acri-Nunes-Miranda & Mondragón-Palomino [12]	*SEP3-like*; partial sequence is available in the paper	XP_020592894.1
	*PeAGL6-2*	XM_020737235.1	GenBank	Xu *et al*. [13]	Named as *PeMADS9* in the paper	XP_020592894.1
	*PeNAC67*	XM_020733068.1	GenBank	Xu *et al*. [13]		XP_020588727.1
	*PeKAN2*	XM_020727309.1	GenBank	Xu *et al*. [13]		XP_020582968.1
	*PeSCL23*	XM_020733890.1	GenBank	Xu *et al*. [13]		XP_020589549.1
	*PaAGL6-2*	PATC138772	Orchidstra 2.0	Su *et al*. [14]		XP_020595851.1
Floral symmetry	*PeCYC1*	KT258891	GenBank	Lin *et al*. [15]		XP_020591342.1
	*PeCYC2*	KT258892	GenBank	Lin *et al*. [15]		XP_020584437.1
Floral color patterning	*PeMYB2*	AIS35919	GenBank	Hsu *et al*. [16]		XP_020582444.1
	*PeMYB12*	AIS35929	GenBank	Hsu *et al*. [16]		XP_020578104.1
	*PebHLH1*	KF769482	GenBank	Hsu *et al*. [16]		XP_020586826.1
	*PeMYC4*	KAM0769787	GenBank	Wang *et al*. [17]		XP_020573641.1
	*PeMYB4L*	XP_020584868.1	GenBank	Wang *et al*. [17]		XP_020584868.1
	*PebHLH*	AIS35934	GenBank	Hsieh *et al*. [18]		XP_020586826.1
	*PeMYB11*	AIS35928	GenBank	Hsu *et al.* [19]		XP_020573611.1
	*PeMYB7*	AIS35924	GenBank	Zhao *et al*. [20]		XP_020599272.1
	*PeMYBx*	WAS06747	GenBank	Fu *et al*. [21]		XP_020578104.1
Floral scent	*PbGDPS*	EU023907	GenBank	Hsiao *et al*. [22]		XP_020598814.1
	*PbTPS3*	MW645240	GenBank	Huang *et al*. [23]		XP_020576698.1
	*PbTPS4*	MW645241	GenBank	Huang *et al*. [23]		XP_020576697.1
Floral scent	*PbTPS5*	MW645242	GenBank	Huang *et al*. [23]		XP_020586098.1
	*PbTPS9*	MW645244	GenBank	Huang *et al*. [23]		XP_020590463.1
	*PbTPS10*	MW645245	GenBank	Huang *et al*. [23]		XP_020590464.1
	*PbABCG1*	MW175288	GenBank	Chang *et al*. [24]		XP_020581075.1
	*PbABCG2*	MW175287	GenBank	Chang *et al*. [24]		XP_020592864.1
	*PbbHLH4*	KY979199	GenBank	Chuang *et al*. [25]		XP_020595212.1
	*PbbHLH6*	Not available		Chuang *et al*. [25]		
	*PbERF1*	Not available		Chuang *et al*. [25]		

**Table 1:** Continued.

**Floral trait involved**	**Gene name**	**Gene ID**	**Database**	**Publication**	**Note**	**ID in GCF_001263595.1**
	*PbNAC1*	KY979200	GenBank	Chuang *et al*. [25]		XP_020597726.1
	*PbGDPS2*	PBTC019238	Orchidstra 2.0	Chuang *et al*. [25]		XP_020570606.1
	*PbbZIP4*	Not available		Chuang *et al*. [26]	Sequence available in the paper	XP_020575916.1
Flower number	*PaWOX3*	Not available		Hsu *et al*. [27]	Sequence available in the paper	XP_020583099.1
	*PaWOX3B*	Not available		Hsu *et al*. [27]	Sequence available in the paper	XP_020583099.1
Axillary bud dormancy	*PeTB1*	Not available		Meng *et al*. [28]		
	*PeTB2*	Not available		Meng *et al*. [28]		
	*PePIN1b*	Not available		Meng *et al*. [28]		
	*PeSLR1*	Not available		Meng *et al*. [28]		
Cuticle production	*PeERF1*	MG948436	GenBank	Lai *et al*. [29]		XP_020578605.1
	*PeCYP86A2*	XM_020732683.1	GenBank	Lai *et al*. [29]		XP_020588342.1
	*PeCYP77A4*	XM_020725159.1	GenBank	Lai *et al*. [29]		XP_020580818.1
	*PeDCR*	XM_020725429.1	GenBank	Lai *et al*. [29]		XP_020581088.1
	*PeGPAT*	XM_020727266.1	GenBank	Lai *et al*. [29]		XP_020582923.1
	*PaERF105*	Not available		Mao *et al*. [30]	Sequence available in the paper	XP_020573826.1
	*PaHAT14*	Not available		Mao *et al*. [30]	Sequence available in the paper	XP_020586415.1
Cuticular wax biosynthesis	*PaMYB9A1*	PAXXG029600	Orchidstra 2.0	Lu *et al*. [31]		
	*PaMYB9A2*	PAXXG123420	Orchidstra 2.0	Lu *et al*. [31]		
Floral longevity	*PeMADS6*	AY678299	GenBank	Tsai *et al*. [32]	*PePI*	XP_020573549.1
	*PeMADS7*	JN983500	GenBank	Hsieh *et al*. [18]	*SEEDSTICK*-like	XP_020591942.1
	*PebZIP*	lcl| Unigene 59946_Pe_fb	OrchidBase 2.0	Hsieh *et al*. [18]	Not accessible anymore	
	*PeHB*	lcl| AECP-22H12	OrchidBase 2.0	Hsieh *et al*. [18]	Not accessible anymore	
	*PaFYF1*	Not available		Chen et *al*. [33]	Sequence available in the paper	XP_020593611.1
	*PaFYF2*	Not available		Chen et *al*. [33]	Sequence available in the paper	XP_020575362.1
Floral size	*PaAAF*	MK674050	GenBank	Chen *et al*. [34]		XP_020594388.1
	*PeCIN8*	KT258889	GenBank	Wu *et al*. [35]		XP_020576817.1
	*PeMADS1*	AF234617	GenBank	Hsieh *et al*. [18]	*AGAMOUS*-like	XP_020575361.1

The corresponding gene IDs in the GCA_001263595.1 assembly (NCBI) were determined by BLAST analysis.

### Floral inflorescence initiation and formation

*Phal.* orchids can be grouped into two types according to flowering regulation: winter and summer flowering species/cultivars. The majority of typical *Phal.* cultivars belong to the winter flowering type, having 3 to 5 years of juvenile phase and requiring low-temperature treatment for 3 to 5 months to flower. Understanding the mechanisms underlying flowering regulation and searching for alternative treatments to induce flowering have been the focus of efforts to lower the production cost for *Phal.* cultivars [36, 37]. Low-temperature (<26°C and <20°C, day and night, respectively) treatment [38] induced biosynthesis of gibberellin acids (GAs) [39], especially that of GA_1_ [36], whereas temperature above 28°C inhibited floral transition potentially due to the rapid biosynthesis flow-through from ‘active’ GA_1_ to ‘inactive’ GA_8_ [37]. Exogenous 6-benzylaminopurine (6-BAP) or benzyladenine (BA) treatments accelerated inflorescence emergence at 20°C to 23°C, whereas no exogenous hormonal treatments (neither GA_3_, GA_4_, GA_7_, BAP, nor BA) could induce inflorescence initiation above 28°C [37, 40], indicating that hormonal treatments mentioned above probably cannot completely substitute for low-temperature induction in high temperature for floral transition in *Phal.* orchids. Exogenous abscisic acid (ABA) application retarded the emergence of flowering shoots, suggesting inhibitory roles of ABA in floral transition [41].

Homologs of *Arabidopsis* flowering time genes in *Phal.* orchids have been cloned and functionally characterized (Fig. 1B), including *PhFT-1* (*FLOWERING LOCUS T*, from a *Phal.* hybrid—Doritaenopsis Tailin Red Angel ‘V31’) [3], *PhapLFY* (*LEAFY*, from *P. aphrodite* subsp. *formosana*) [4], *ORAP11* and *ORAP13* (*AP1*-like genes from *Phal.* hybrida cv. Formosa rose) [5] and *PhalCOL1* (*CONSTANS-like 1*) [6]. These genes had relatively conserved functions in floral induction as their counterparts in *Arabidopsis*. For example, transgenic tobaccos over-expressing either *ORAP11* or *ORAP13* flowered earlier than wild-type plants [5]. However, *PaFVE* (*flowering locus VE*-like gene from *P. aphrodite* subsp*. formosana*) regulated floral organ maturation in *Phal.* orchids, rather than floral initiation as in *Arabidopsis* [7]. Two J-domain proteins, PhJ3a and PhJ3b (from *Phal.* cultivar ‘Xiao Xue’), potentially promoted flowering by upregulating expression of *PhFT3* while repressing expression of *PhSVP* (*SHORT VEGETATIVE PHASE*) [8]. Transgenic materials overexpressing the *PaFT* gene driven by the ubiquitin promoter had been created, in which activation of *PaFT* and repression of a vegetative gene *Phal. Homeobox1* (*POH1*) were observed [9]. However, the effect of *PaFT* overexpression on inflorescence initiation without cold treatment is still unclear. Nonetheless, studies in the related genus *Dendrobium* demonstrate the feasibility of this approach. For instance, transgenic *Dendrobium* orchids expressing 35S:*DOSOC1* flowered earlier than wild-type plants [42]. This indicates that genetically modifying *Phal*. orchids, e.g. by overexpressing *PaFT*, could potentially shorten their juvenile phase and lower production costs.

### Floral organ identity determination

**Figure 2:**
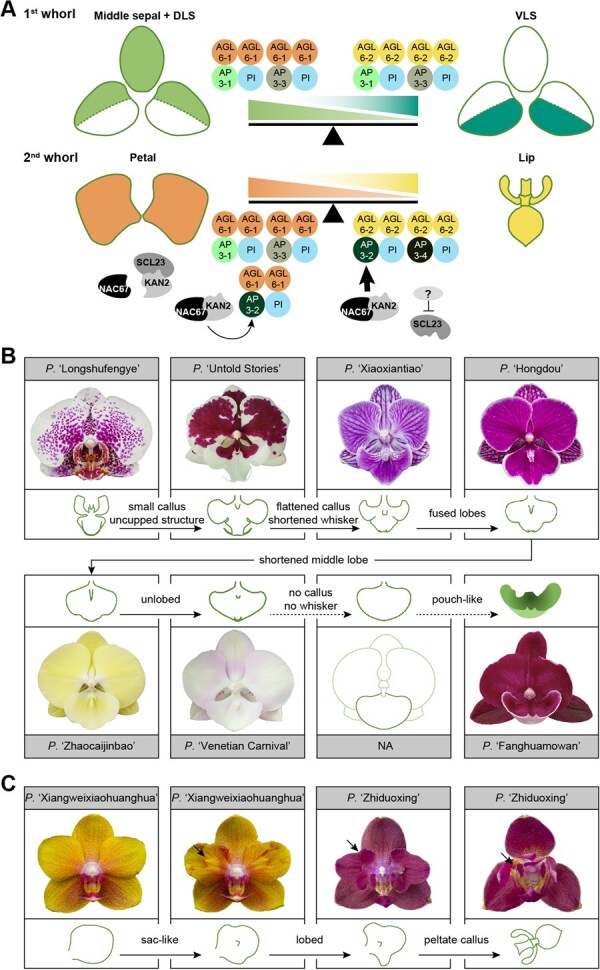
Floral organ identity program in *Phal*. orchids. (A) Diagram for floral organ identity determination, inferred according to refs [13, 44, 46]. *PeAGL6-2* was specifically expressed in the lip and the ventral region of lateral sepals (VLS); *PeAP3-2* was specific to petals and the lip; and *PeAP3-4* was exclusive to the lip. In contrast, *PeAGL6-1*, *PeAP3-1*, *PeAP3-3* and *PePI* are broadly expressed across all floral organs. KAN2 and NAC67 form a complex in the lip to promote *PeAP3-2* expression, while SCL23 competes with NAC67 for interaction with KAN2 in petals, thereby disrupting the activation of *PeAP3-2*. A set of four circles denotes a tetrameric complex of MADS-box proteins. DLS: dorsal region of lateral sepals, respectively. *AP3*: *APETALA3*; *AGL6*: *AGAMOUS-LIKE 6*; *PI*: *PISTILLATA*; *SCL*: *STEM CELL LEUKEMIA*; *KAN*: *KANADI*; *NAC*: *NAM, ATAF1/2, and CUC2*. Thick and thin arrows: strong and weak activation, respectively; line with blunt end: repression. (B) A series of *Phal.* orchids showing different lip shapes. *P.* ‘Longshufengye’ has a normal lip; lip of *P.* ‘Untold Stories’ is uncupped and with smaller callus, but with three lobes and whiskers unchanged; lip of *P.* ‘Xiaoxiantiao’ has a flattened callus, shortened whiskers and three lobes; lip of *P.* ‘Hongdou’ has fused lobes and even shorter whiskers; lip of *P.* ‘Zhaocaijinbao’ has a shortened middle lobe; lip of *P.* ‘Venetian Carnival’ is unlobed; a complete petal-like organ should be flat, unlobed, without calluses and whiskers; lip of *P.* ‘Fanghuamowan is a pouch-like organ without lobes and calluses, but with two short whisker-like structures. NA: not available. (C) Mutants of *P.* ‘Xiangweixiaohuanghua’ and *P.* ‘Zhiduoxing’ showing different types of P-to-L transformation. Arrows indicate sac-like structure, obvious lobes, and peltate callus.

Flowers of *Phal.* orchids typically have one middle (dorsal) and two lateral (ventral) sepals, two lateral petals, one lip, one gynostemium containing two (only in subgenus *Phalaenopsis*) or four pollinia, and ovules embedded in the ovary (Fig. 1A and B). In flowering plants, the floral organ identities are generally determined by the MIKC^C^-type MADS-box genes, which function in a frame known as ABCDE model, where A + E genes determine the identity of sepals, A + B + E, B + C + E, C + E, and C + D + E determine the identities of petals, stamens, carpels, and ovules, respectively [43]. There were several modified models proposed to explain the formation and diversification of perianth in orchid flowers, such as the ‘Orchid Code’ model [44], ‘Homeotic Orchid Tepal’ (HOT) model [45], and ‘Perianth code’ (P code) model [16]. The ‘Orchid Code’ and HOT models were summarized according to expression profiles of four *AP3* (*APETALA3*, B class)-like genes in *Phal.* orchids (Fig. 1C), where *PeMADS2* (corresponding to *PeAP3-1*) and *PeMADS5* (*PeAP3-3*) were expressed in all the perianth, *PeMADS3* (*PeAP3-2*) was expressed in petals and lip, *PeMADS4* (*PeAP3-4*) was specifically expressed in the lip (Fig. 2A). The P code model was inferred according to different expression profiles of *OAP3-1* (*OMADS5*, ortholog of *PeMADS2*), *OAP3-2* (*OMADS9*, ortholog of *PeMADS3*), *OAGL6-1* (*AGAMOUS-LIKE 6-1*, *OMADS7*), *OAGL6-2* (*OMADS1*), and *OPI* (*PISTILLATA*, *OMADS8*) in the perianth of several orchid species, where *AGL6-1/2* are G class genes which are not involved in floral organ identity determination in *Arabidopsis*. According to the P code model, the formation of sepal/petal and lip was determined by the competition between SP (AP3-1/AGL6-1/AGL6-1/PI) and L (AP3-2/AGL6-2/AGL6-2/PI) complexes (Fig. 2A) [16, 46]. Functions of E class genes, *PeSEPs* (*SEPALLATA*), were potentially conserved in orchids as in other flowering plants: silencing *PeSEPs* led to the transformation of perianth into leaf-like organs [20]. Specifically, *PhaMADS7* (*SEP3*-like from *Phal.* hybrid ‘Athens’) was expressed at the highest levels in lip and lip-like petals in peloric *Phal.* flowers, indicating its role in lip development [12].

Two types of floral mutants with homeotic transformation between different organ types are commonly seen in *Phal.* orchids: flowers with petal-like lips (L-to-P, ‘Bigfoot’) or lip-like petals (P-to-L). For the L-to-P transformation, silencing *PeAGL6-2* via virus-induced gene silencing (VIGS) led to petal-like organs [16, 46], which were more likely uncupped lips with the basic lip structures unaffected (three lobes, two whiskers, and one callus). However, the lip shapes in ‘Bigfoot’ cultivars range from this type of uncupped lips to near-complete petal-like organs (Fig. 2B). Since the VIGS technique only knocks down, rather than knocks out, expression of *PeAGL6-2*, it remains unclear whether the lips would be transformed into complete petal-like organs if expression of *PeAGL6-2* was totally reduced in wild type orchids, and whether these petal-like organs are results of loss-of-function of *PeAGL6-2* in these ‘Bigfoot’ cultivars. Knock-out or gene editing of *PeAGL6-2* is required to test these hypotheses.

**Figure 3:**
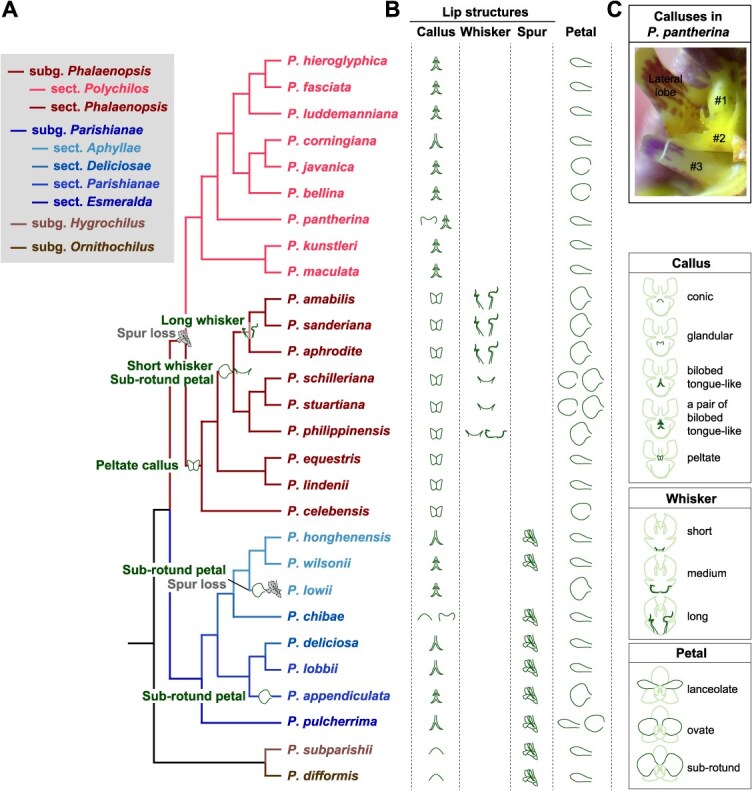
Emergence and evolution of four floral traits in *Phal.* orchids. (A) The phylogeny of *Phal.* orchids was inferred from ref [47], where the subgenus and sections were inferred from ref [1]. Evolutionary events are marked on the phylogeny, including the loss of spurs, as well as the emergence of short and long whiskers, sub-rotund petal shape, and peltrate callus. (B) Shapes indicate the presence of different lip structures (left three panels) and the petal shape (rightmost panel)—as illustrated by the legends on the right, in each species. (C) Three calluses (numbered ‘#1,’ ‘#2,’ and ‘#3,’ respectively) in *P. pantherina.*

Interestingly, along with different L-to-P degrees, characteristics of lips disappear sequentially (Fig. 2B): cupped shape of three lobes, thick peltate callus and long whiskers, notches between three lobes, and three lobes. Structures of these intermediate petal-like lips mimic those in other *Phal.* species or orchids outside the genus *Phal.* For example, flattened calluses (e.g. *P.* ‘Zhaocaijinbao’) resemble those in species outside the section *Phalaenopsis* (e.g. *P. appendiculata*); the trilobed lips without notches (e.g. *P.* ‘Hongdou’ and *P.* ‘Zhaocaijinbao’) resemble the middle lobes of *P. appendiculata* and *P. pulcherrima*. Some of those lip characteristics arose sequentially along with the evolutionary history of species in the section *Phalaenopsis* (Fig. 3A). Particularly, peltate callus arose in the most recent common ancestor (MRCA) of the section *Phalaenopsis*; whiskers appeared after the split between *P. equestris*/*P. lindenii* and other species, and longer whiskers only arose in the MRCA of *P. amabilis*, *P. sanderiana* and *P. aphrodite*. In addition, the lip of a hybrid progeny in our greenhouse is pouch-like with no calluses (named as *P.* ‘Fanghuamowan,’ lower right panel in Fig. 2B), which is similar to the slipper in *Paphiopedilum* and *Cypripedium* orchid flowers. Pouch-like structures or spurs are ancestral traits for *Phal.* orchids and have been lost from MRCA of species in subgenus *Phalaenopsis* (Fig. 3A). These observations suggest the formation of lips in *Phal.* orchids may involve a series of development repatterning events during the evolution of *Phal.* orchids, similar to the evolution of elaborate petals in *Nigella* (Ranunculaceae) species [48].

P-to-L transformation has been observed independently for several *Phal.* species and cultivars, such as *P. equestris* [13, 49], *Phal.* hybrid ‘Athens’ [12], *P.* ‘KHM190’ [50], and three other cultivars in Chen *et al.* [51] P-to-L transformation shows different degrees as well (Fig. 2C). Tsai *et al*. [10] identified three insertions in the upstream region and fifth intron of both *PeMADS5* alleles, and found no *PeMADS5* expression in *P. equestris* P-to-L peloria compared with in normal flowers. Thus, they proposed that these insertions might be the potential cause leading to the defective expression of *PeMADS5* and the P-to-L transformation in *P. equestris*. However, silencing *PeMADS5* did not produce lip-like petals [52], suggesting that further validations are still required to confirm the underlying causes of the P-to-L transformation in these peloria. Chen *et al*. [51] identified differential gene expressions between normal and P-to-L flower buds, including up-regulated expressions of *bZIP* (*basic leucine zipper*)-like genes, *Teosinte branched1/Cincinnata/proliferating cell factor* (*TCP*)-like genes, auxin-regulated protein kinase and cyclophilin related genes, and down-regulated expressions of genes involved in DNA methylation, chromatin remodelling and post-transcriptional regulation. Xu *et al*. [13] found that in the lip-like petals of *P. equestris* peloria, *PeNAC67* (*NAM, ATAF1/2, and CUC2*) and *PeSCL23* (*STEM CELL LEUKEMIA*) were expressed at higher levels compared with in the lips. Silencing *PeNAC67* led to reverse conversion of lip-like petals to normal petals, whereas silencing *PeSCL23* led to even stronger P-to-L transformation. PeKAN2 (KANADI) functioned together with PeNAC67 to activate the L complex, while PeSCL23 interacted with PeKAN2 to compete with the L complex thus promoting petal formation [13] (Fig. 2A). Although those studies have enhanced our understanding about the differences in genetic programs underlying petal and lip formations, changes of gene expression in these genetic programs were more likely results, rather than causes, of the P-to-L transformation. Further thorough investigation is needed to uncover the genetic changes giving rise to the P-to-L peloria in different *Phal*. orchids.

### Floral symmetry determination

In *Antirrhinum majus* L., the dorsoventral asymmetry of flowers is determined by the synergetic interactions among *TCP*-like genes *CYCLOYDEA* (*CYC*) and *DICHOTOMA* (*DICH*), *MYB*-like genes *DIVARICATA* (*DIV*), *RADIALIS* (*RAD*), as well as *DIV-*and*-RAD-*interacting-factor (*DRIF*) [53–57]. Specifically, *CYC/DICH* positively regulate *RAD* expression in the dorsal region, and *RAD* prevents the formation of *DRIF-DIV* heterodimers by interacting with *DRIF* in the dorsal region; *DIV* is essential for the ventral development. This zygomorphy determination program is relatively conserved in core eudicots, and the dorsal-specific *TCP* genes (*CYC*, *DICH*, and *Arabidopsis TCP1*) originated from gene duplications that had occurred within core eudicots, thus it was proposed that there were no true orthologs of *CYC*/*DICH* outside core eudicots [56]. In Commelinaceae and Poaceae, it was proposed that the zygomorphy was determined by the dorsoventrally differential expression of *TCP*-like genes and B-type MADS-box genes [58, 59].

In orchids, expression of *DIV*-like, *RAD*-like and *DRIF*-like genes were higher in lip than in petals, whereas in P-to-L flowers, their expression levels were similar between lip and transformed petals [60], indicating these three genes may participate in the determination of zygomorphy in orchids. Expressions of *PeCYC1* and *PeCYC2* were also higher in lateral sepals and lip than in dorsal sepal and petals [15]; however, their expression profiles were not associated with flower morphologies in *Cymbidium sinense* varieties with different floral symmetries [61]. Instead, Su *et al*. [61] found association between floral symmetry and *CsAP3-2* and *CsAGL6-2* expression, suggesting that B- and G-class genes might be crucial for floral zygomorphy in *Cymbidium* orchids and potentially those in other orchids including *Phal.* as well. Expression of *PeMADS4* (*PeAP3-4*) [45] and *PaAGL6-2* (named as *PaAGL6-1* in ref [14]), which were lip-specific and ventral region-specific in normal *Phal.* flowers, respectively, were expanded to the transformed petals in P-to-L flowers. These results indicate that *MYB* (e.g. *DIV*, *RAD*, *DRIF*) and MADS-box genes (B- and G-classes) might cooperate to determine the zygomorphy of orchid flowers (Fig. 1C), and the transition from zygomorphy to actinomorphy in P-to-L flowers is likely caused by genetic or epigenetic changes that led to the upregulation of these genes in the dorsal region.

### Floral pigments and color patterning formation

**Figure 4:**
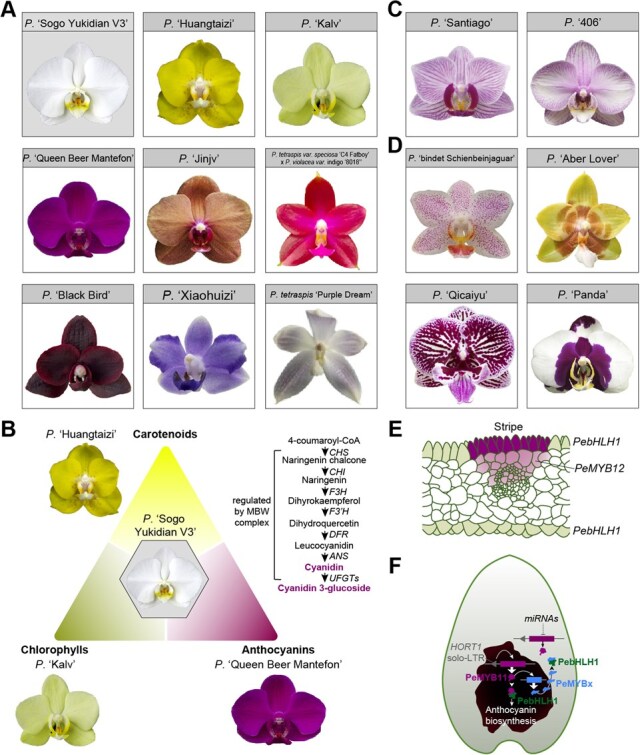
Diversity of floral color patterning in *Phal*. orchids and underlying genetic mechanisms. (A) *Phal.* orchids with different colors. (B) The different content ratios among chlorophylls, carotenoids and anthocyanins contribute to the formation of green, yellow, pink and intermediate color types in *Phal*. orchids. The pathway for cyanidin biosynthesis—one of the most predominant anthocyanins in *Phal*. orchids—is shown on the right. MBW complex: complex formed by R2R3-MYB, basic helix–loop–helix (bHLH) transcript factors and Tryptophan-aspartate 40 repeat-containing (WD40 repeat) proteins. *CHS*: *chalcone synthase*; *CHI*: *chalcone isomerase*; *F3H*: *flavanone 3-hydroxylase*; *F3’H*: *flavonoid 3′-hydroxylase*; *DFR*: *dihydroflavonol-4-reductase*; *ANS*: *anthocyanin synthetase*; *UFGTs*: *uridine diphosphate-glucose: flavonoid 3-O-glucosyltransferas*. (C, D) Different stripe (C) and spot (D) patternings in *Phal.* orchids. (E) Diagram for stripe formation in *Phal*. orchids, inferred from refs [62, 63], where the formation of venation-patterned stripes is driven by the specific expression of *PebHLH1* in epidermal cells and *PeMYB12* above the vasculature. (F) Diagram for spot formation in *Phal*. orchids, inferred from refs [20, 21, 64, 65]. *HORT1* solo-LTR enhances expression of *PeMYB11*, which activates expression of *PeMYBx* and the anthocyanin biosynthesis within the spot/patch region. PeMYBx protein moves to neighboring background cells and forms complexes with PebHLH1 thus inhibits PeMYB11’s function and the anthocyanin biosynthesis. Expression of *PeMYB11* is repressed by *miRNAs* in the background cells as well. Thin arrow: activation; lines with blunt ends: repression; wide arrow: translation; arrow head: complex formation.

Flowers of *Phal.* orchids display an extensive range of colors, including white, yellow, light green, orange, pink, red, dark red, purple-blue, grey, and so on (Fig. 4A). Green, yellow, and pink flowers tended to have higher contents of chlorophylls, carotenoids, and anthocyanins, respectively [66] (Fig. 4B). Cyanidin-based anthocyanin was found to be the predominant contributor to red-purple, purple, purple-violet, violet and violet-blue colors in *Phal.* orchids [67]. The anthocyanins are produced by a series of reactions starting from 4-coumaroyl-CoA, catalysed by structural enzymes such as chalcone synthase and anthocyanin synthetase [68]. Functions of structural enzymes in anthocyanin biosynthesis were shown to be conserved in *Phal.* orchids [20, 69, 70]. R2R3-MYB and basic helix–loop–helix (bHLH) transcription factors interact with Tryptophan-aspartate 40 repeat-containing (WD40 repeat, or WDR) proteins to form MBW complexes and regulate the biosynthesis of anthocyanins [71]. *PeMYB2* and *PeMYB12* were reported to determine the pink color formation for sepal/petal and lip, respectively [62]. *PeMYB4L* also promotes anthocyanin accumulation and red color formation in *Phal.* orchids, while bHLH protein PeMYC4 forms complex with PeMYB4L to repress anthocyanin accumulation [17]. Silencing *PebHLH* resulted in the bleaching of red color in the dorsal region of lip lateral lobes [18], indicating that *PebHLH* might be activated by dorsal programs in the lip, and there should be another *bHLH* copy participating in color formation in the ventral region.

Beside diverse colors, *Phal.* orchid flowers also show extensive diversity in color patterning, including stripes and spots (Fig. 4C and D). Stripes on *Phal.* orchid flowers generally show venation patterning (Fig. 4C), which is commonly seen in other flowering plants, such as *Crocus vernus* (Iridaceae), *Cryptostylis erecta* (Orchidaceae), and *Veronica chamaedrys* (Plantaginaceae). In *A. majus* L. lines AA114, the epidermal-specific venation was produced by overlapping expression domains of *R2R3-MYB* gene *Venosa* (expressed in a wedge of cells radiating from the vasculature) and *bHLH* gene *DELILA* (expressed specifically in epidermal cells) [63]. In *Phal.* orchids, *PeMYB12* was shown to determine the venation formation on the sepals/petals, besides its proposed function in color formation for the lip [62]. Expression profiles of *PeMYB12* and *PebHLH1* were similar to those of *Venosa* and *DELILA* as well [62], indicative of the conservation of venation-patterned stripe formation (Fig. 4E). Silencing *OAGL6-1*, *OAP3-1,* and *OPI* led to the erasion of venation on sepals/petals with their shapes remaining unchanged, suggesting that *OAGL6-1*, *OAP3-1,* and *OPI* may function upstream of *PeMYB12* in venation determination [46].

The most prominent and diverse pigment characteristics of *Phal.* flowers are the presence/absence (P/A) and types of spots (Fig. 4D). *PeMYB11* was proposed to determine the spot formation [62] and function downstream of *PeAGL6-2* [46]. However, silencing *PeMYB11* did not affect spots on the callus [46], suggesting that the formation of these spots is potentially governed by regulators distinct from *PeMYB11*. The insertion of one long terminal repeat (solo-LTR) of *Harlequin Orchid RetroTransposon 1* (*HORT1*) in the upstream regulatory region of *PeMYB11* in *P.* ‘Yushan Little Pearl’ enhanced *PeMYB11* expression [19]. This led to extremely high accumulation of anthocyanins and the formation of purple patches. On the other hand, the insertion of full-length *HORT1*, containing two LTRs and the coding DNA sequence region, resulted in decreased expression of *PeMYB11* [19]. *PeMYB7* was also shown to be associated with the purple patch formation in *P.* ‘Panda,’ and *microRNA156g* (*miR156g*) and *miR858* targeted and downregulated expression of *PeMYB7* and *PeMYB11* in the non-spot area, respectively [20].

The formation of diverse spots in *Phal.* orchids can be explained using an activator-inhibitor system, which was first proposed to interpret the spot formation in monkey flowers (*Mimulus lewisii*, Phrymaceae) [64]. *M. lewisii* has dispersed pink spots on the yellow nectar guides of the ventral petal. *R2R3-MYB* gene *NECTAR GUIDE ANTHOCYANIN* (*NEGAN*) activates anthocyanin biosynthesis and the expression of *R3-MYB* gene *RED TONGUE* (*RTO*) in spots. Then the mobile RTO protein moves to neighboring cells and suppresses NEGAN’s activity by competing for its bHLH protein partners [64]. This activator–inhibitor system of *R2R3-MYB* and *R3-MYB* genes is potentially conserved in *Phal.* orchids (Fig. 4F). Supporting this, both *R2R3-MYB* gene *PeMYB11* and *R3-MYB* gene *PeMYBx* (repressor of anthocyanin biosynthesis) were expressed specifically in the purple patches but not in the adjacent white background regions [21]. Moreover, simulating *PeMYB11* and *PeMYBx* expression using the activator-inhibitor system mimics the dispersed and rounded spots [19, 65]. Successively introducing the effects of *HORT1* solo-LTR, *miR858* and full-length *HORT1* on *PeMYB11* expression in the simulation resembled different spot/patch distributions in *Phal.* cultivars. Particularly, integrating these above factors successfully simulated patch patterning of *P.* ‘Yushan Little Pearl’ [65], supporting the proposed complex determination program of spot/patch formation in *Phal.* orchids.

According to the phylogeny of *MYB* genes in Hsu *et al*. [62], *PeMYB2*, *PeMYB11,* and *PeMYB12* were produced by recent duplications which may be orchid-specific. The extremely complicated color patterning in *Phal.* orchids is potentially due to frequent gene duplications of *MYB* genes, quick sequence divergences and sub-/neo-functionalization among duplicates, interplays between *microRNAs* and *MYB* genes, between *R2R3*- and *R3*-*MYB* genes, and between *MYB* and MADS-box genes.

### Floral scent formation

Floral scent of *Phal.* orchids plays vital roles in attracting pollinators and defense. It has become a key horticultural trait in breeding programs as well. The predominant volatiles in most fragrant *Phal.* orchids are monoterpenes, such as linalool, geraniol and their derivatives [72–74]. In *P. stobartiana*, however, the floral scent is characterized by a distinct profile dominated by benzenoids and nitrogen-containing compounds [74]. No monoterpenes were detected in the scentless flowers of *P. equestris* and *P. aphrodite* [25, 72]. *Phal.* flowers release their fragrance from the first day of flowering, rapidly increase the emission during flower maturation with peaks at the full-bloom stages (4–8 days after flowering), and then gradually decrease the emission later on [25, 75]. During the day, *P. schilleriana* and *P. bellina* release the highest amount of volatiles in the morning when their pollinators are active [75, 76]. Chuang *et al*. [77] found that under constant light, *P. violacea* flowers emitted monoterpenes in a diurnal pattern, whereas constant darkness suppressed their scent emission. Promoters of the structural genes and transcription factors responsible for monoterpene biosynthesis harbored light- and circadian-responsive elements, suggesting their diurnal expression is regulated by environmental light and the internal circadian clock. Chen *et al*. [74] found associations between volatile compounds with morphological traits—including petal shape, flower color, plant height, and width—in *Phal.* species/cultivars. These associations point to either the coinheritance or mutually antagonistic biological activities between these different traits.

*P. bellina* GERANYL DIPHOSPHATE SYNTHASE (PbGDPS) played as the key enzyme for the biosynthesis of geranyl diphosphate that is the precursor of monoterpenes [22]. *PbTPS3* (*terpene synthase*), *PbTPS4*, *PbTPS5*, and *PbTPS10* were responsible for the production of linalool, *PbTPS5* and *PbTPS9* were involved in the biosynthesis of geraniol, and PbTPS3 catalysed the biosynthesis of (β)-cis-ocimene [23]. Two ABC subfamily G genes *PbABCG1* and *PbABCG2* played substantial roles in the transport of monoterpene and the emission of scent in *P. bellina* [24]. Transcription factors PbbHLH4, PbbHLH6, PbbZIP4, PbERF1 (APETALA2/ethylene responsive factor), and PbNAC1 facultatively or obligatorily bind to promoter sequences of *PbGDPS*, *PbGDPS2*, *PbTPS5,* and *PbTPS10* to positively regulate their expression [25]. In addition, expression of *PbGDPS* and *PbbHLH4* were closely associated with the monoterpene production in *Phal.* orchids [25]. Chuang *et al*. [26] identified two repeat sequences in the *PbGDPS* promoter region (*PbGDPSp*) that were crucial for its activity, and found that scentless *Phal.* species had truncations in one or both repeats. Furthermore, they proposed PbbZIP4 as the transcription factor that binds to this dual-repeat promoter to activate *PbGDPS* expression. Consistently, monoterpene production was exclusive to *Phal.* orchids that harbored both *bZIP4* expression and the intact dual-repeat *GDPS* promoter [26]. These findings indicate differences in both *cis*- and *trans*-regulation between fragrant and scentless *Phal.* flowers and explain the difficulties for creating fragrant *Phal.* cultivars via cross breeding.

### Determination of other miscellaneous floral traits that are important in industrialization

#### Inflorescence-related traits (lignification, number, floral bud count per inflorescence and axillary bud dormancy)

Most raceme-type large-flowered *Phal.* cultivars require sticking to support the inflorescences, increasing labor costs and making the breeding for erect/sub-erect inflorescences a key objective. In addition, cultivars with multiple inflorescences bolting simultaneously and ‘dragon orchids’ (with ≥18 simultaneous open flowers per raceme) have become very popular in recent years and have considerable economic value. Panicle-type cultivars with small-to-medium flowers and multiple branching are gaining popularity as well. Erect and sub-erect inflorescences tended to have higher proportion of lignified area per total stem area and thicker lignified fiber walls in the peduncle than arching and pendant inflorescences [78]. The positive correlation between similarities in inflorescence lignification variables and phylogenetic relationships among *Phal.* orchids suggests that the degree of lignification might be heritable [78]. Existing *Phal.* cultivars normally have one to three inflorescences, and exogenous application with 6-BAP or BA during the flowering forcing stage can increase the inflorescence numbers [37, 40]. However, it remains largely unexplored what genetic mechanisms contribute to the inflorescence lignification and inflorescence numbers in *Phal.* orchids.

To produce ‘dragon orchids’, it typically requires extending the juvenile phases for additional more than one year prior to the induction of the first inflorescence. Leaf carbon-to-nitrogen (C/N) ratio [79] and potassium (K) concentration in the culture media [80] were found to have positive contribution to flower counts. Plants grown under lower temperatures tended to have more flower buds on the first inflorescence than those under higher temperatures, but with no significantly longer inflorescence length [38]. Two *WUSCHEL*-related homeobox genes, *PaWOX3* and *PaWOX3B*, participated in flower initiation, as *PaWOX3-* and *PaWOX3B-*silenced flowers had significantly reduced floral bud numbers but with the floral morphology unchanged [27].

A typical inflorescence possesses three to six dormant buds at its base, which are utilized for the clonal propagation of *Phal*. orchids through tissue culture. In raceme-type cultivars, decapitation treatment often induces the top one or two dormant buds to develop into new floral branches, likely due to the removal of apical dominance. In contrast, panicle-type cultivars naturally develop branches from buds at corresponding positions without requiring decapitation. In the raceme-type cultivar *Phal.* ‘Big Chilli’, two class II *TCP* genes, *PeTB1/2* (homologs of *TEOSINTE BRANCHED 1*), function as key regulators of axillary bud dormancy by repressing local *PePIN1b* expression [28]. Moreover, the DELLA protein PeSLR1 (a homolog of rice Slender Rice 1) interacts with PeTB1 to enhance its repression on *PePIN1b* expression, which is an auxin efflux carrier promoting axillary bud development [28]. However, the regulatory mechanisms controlling lateral floral branch development in panicle-type cultivars remain unexplored. Understanding the differences in bud dormancy programs between raceme- and panicle-type cultivars is crucial for developing novel, large-flowered, panicle-type cultivars.

#### Cuticle/epidermal cell/cuticular wax biosynthesis traits

Cuticular wax on the parianth epidermal cells in *Phal.* plays important roles in biotic and abiotic stresses, and moisture conservation, which potentially be an important reason for the long flowering phase in *Phal.* orchids. Parianth of *Phal.* orchids can be waxy and velvety, where waxy petals have flat epidermal cells that are tightly arranged, relatively thick and smooth cuticles, while velvety petals have conical epidermal cells that are loosely arranged, and thin cuticles [81]. In some *Phal.* orchids with velvety parianth, the lip exhibits epidermal cells with heavier cuticles compared with sepals and petals [11]. Silencing *PeSEP3* reduced the cuticular folding in the lip epidermis, potentially due to the downregulation of an *AP2*/*EREBP*-like gene [11]. The *AP2*/*ERF* gene *PeERF1* promotes the formation of nanoridges of lips probably by upregulating the potential cutin biosynthesis genes, such as *PeCYP86A2* (*cytochrome P450*), *PeCYP77A4*, *PeDCR* (*defective in cuticular ridges*), and *PeGPAT* (*glycerol-3-phosphate acyltransferase 6*) [29]. However, *PaERF105* (*DECREASE WAX BIOSYNTHESIS 2*-like) functions as repressor of cuticle production, and the homeodomain-leucine zipper II gene *PaHAT14* represses *PaERF105* expression thus promotes cuticle deposition during the early flowering stages [30]. Two *R2R3-MYB* genes *PaMYB9A1* and *PaMYB9A2* function in the development of conical epidermal cells and cuticular wax biosynthesis [31].

#### Floral longevity

Long flowering phase of *Phal.* orchids is a core industrial advantage, which makes characterizing the underlying mechanisms essential for further extending their commercial longevity. The senescence begins 1 to 2 days post-pollination, due to the pollination-induced increase in ethylene sensitivity [82]. MIKC^C^-type MADS-box genes were reported to suppress flower senescence. Overexpressing *PeMADS6* (*PePI*) in *Arabidopsis* resulted in petaloid sepals and a 3- to 4-fold increase in flower longevity [32]. Silencing *PeMADS7* (*SEEDSTICK*-like) caused the abortion of floral buds [18]. Silencing *OAP3-1* and *OPI* promoted sepal/petal senescence in *Phal.* orchids [46]. *PaFYF1/2* (*FOREVER YOUNG FLOWER*, *AGAMOUS*-like gene, from *P.* 'sogo yukidian V3') potentially prohibit floral senescence/abscission by suppressing ethylene signaling and abscission-associated genes [33]. Furthermore, silencing *PebZIP* and *PeHB* (*homeobox*, potentially co-silenced with its paralogous genes) also resulted in floral abortion, suggesting their functions in suppressing flower senescence as well [18].

#### Floral size

Floral size of *Phal.* orchids varies widely and is a key breeding target as well. *Phal.* cultivars with monopodial and large flowers are normally used for artistic pot display during festivals, and tend to be tetraploids or near-tetraploids, whereas those with small and medium flowers exhibited a more diverse range of chromosome numbers [83, 84]. Floral size was also found to be positively associated with the leaf C/N ratio [79] and K concentration in the culture media [80], indicating effects of nutrition conditions during the juvenile phase on floral sizes. Endoreduplication was found to contribute to floral sizes in *Phal.* orchids as well: (i) large and medium flowers tended to have larger petal cell sizes and higher endoreduplication levels than small ones, particularly in proximal regions of petals; (ii) the endoreduplication levels increased during flower development [85]. Among other floral organs, petal sizes directly contribute to flower sizes in *Phal.* orchids, and the morphogenesis of petals involves cell division till flower development stage 2 and rapid expansion during stages 3 to 5 [86]. A recent review paper has provided an overview of current advances in understanding the regulation of petal sizes in chrysanthemums and orchids [87]. In summary for *Phal.* orchids, *PebHLH*-silenced and *PeMADS1*-silenced flowers were significantly smaller than mock-treated flowers, potentially due to premature termination of cell proliferation and reduced cell expansion, respectively, during the flower development [18]. Larger flowers in *Phal.* cultivars were potentially associated with higher *PaAAF* (*Auxin Activation Factor*) expression, more and larger cells in the perianth [34]. In addition, *Phal.* cultivars with larger flowers tended to have higher *PeCIN8* (*CINCINNATA*) gene expression and weaker resistance to yellow leaf disease [35].

#### Floral organ shape and structure

All the floral organs differ in shapes among different *Phal.* species/cultivars. For example, petal shapes in *Phal.* orchids can be roughly grouped into three classes according to their aspect ratios: lanceolate, elliptic or ovate, and sub-rotund. The majority of *Phal.* cultivars with large flowers have sub-rotund petals, which might have arisen independently in the MRCA leading to *P. amabilis* and *P. schilleriana*, in *P. lowii* and in *P. appendiculata* (Fig. 3A). Molecular mechanisms underlying the diversification in petal shapes remain largely unexplored. Silencing *PeMADS6* caused the transformation of petals from sub-rotund to triangle-like and with wrinkled edges [46], indicating the sub-rotund shape of petals potentially requires full function of *PeMADS6*.

The lip has the most complicated structures in *Phal.* orchids, and may have three or five lobes (e.g. middle lobe of *P. pulcherrima* is further trilobed), whiskers, one to three calluses, spur or sac-like structures, keels, filamentous appendages, hairs, clawed lobes and so on. These structures also show high diversity in P/A, shapes and sizes among *Phal.* orchids. The simplified categories of calluses in Fig. 3B can barely display the callus diversity, particularly those in the section *Polychilos*. For example, there are three calluses with totally different shapes in *P. pantherina* (Fig. 3C). Gynostemium in *Phal.* orchids may contain coarsely erose-dentate hood (e.g. *P. corningiana*) or long proboscis-like hoop (e.g. *P. lowii* and *P. zhejiangensis*) over the pollinia, with/without stelidia at the base, and mentum that is a spur-like outgrowth connecting the gynostemium and lip. Callus, stelidia, and mentum play important roles in pollination, by providing a holdfast for the pollinating insects, trapping their head or other body parts, and acting as a hinge to slam the insects against the anther and stigma, respectively [88]. The micro 3D-CT scan and gene expression analysis indicated that callus has a mixed petaloid–staminodial origin, stelidia is of staminodial origin and mentum is of mixed sepaloid–petaloid–staminodial origin [88]. More investigations should be conducted on the genetic mechanisms underlying the formation and diversification of these structures.

### Future perspectives

Potentially due to the difficulty in obtaining sufficient seedlings of wild *Phal.* species, previous studies mainly used commercial cultivars to investigate the genetic mechanisms underlying floral trait formation and diversification, which had complex genetic backgrounds. These studies generally overlooked the questions of how these horticultural characteristics arose in or were introduced into *Phal.* orchids, and how they differed and evolved in *Phal.* orchids. Addressing these questions requires reliable evolutionary relationships among wild *Phal.* species and cultivars, along with elaborate morphological description, classification and quantization. In Fig. 3, we mapped some of the floral traits onto a simplified phylogeny inferred from a study utilizing sequences of nuclear ribosomal DNA internal transcribed spaces and three plastid DNA sequences [47]. However, this is far from sufficient to capture the high diversity of floral traits in *Phal.* orchids, particularly for the extremely complicated lip structures and their shapes. Genome sequencing or resequencing of wild *Phal.* species—possibly including related hybrid parent species outside the genus—as well as cultivars, could help clarify species relationships and trace the parental origins of cultivated varieties. However, the two currently available reference genomes (*P. equestris* and *P. aphrodite*, both scentless) are based on short-read sequencing technology and remain at the scaffold levels [89, 90].

Furthermore, due to the relatively weak gene silencing phenotypes of current VIGS techniques, high allelic heterogeneity, and potential gene redundancy in *Phal.* orchids, the functions of some previously reported genes remain incompletely characterized. For example, although several models have been proposed for perianth identity determination in *Phal.* orchids, complete homeotic transformation between floral organs types via VIGS have not been archived, except for the transformation from sepals to leaf-like organs in *PeSEP3*-silenced flowers [11]. Knock-out or gene editing of target genes via genetic transformation is essential to obtain loss-of-function mutants of these genes to validate their functions. Although several studies have reported successful genetic transformation in *Phal.* orchids, as reviewed in Zhang *et al*. and Enoki and Takahara [91, 92], currently, it still lacks a stable and efficient transformation system. These limitations hamper the development of genomics-assisted and molecular breeding in *Phal*. orchids.

To address the constraints noted above, the following strategies are proposed. First, genome-wide association study (GWAS) analysis can be employed as an efficient approach to associate genetic and phenotypic variations, with minimal reliance on wild-type plant materials. This is particularly crucial for traits that are absent in those two sequenced species, such as floral scent, spur, and other types of calluses (Fig. 3). Thus, this strategy requires a reference pangenome that captures the collective genetic diversity of major *Phal.* orchids, particularly those used in *Phal.* breeding. Pangenomes have been shown to capture missing heritability and enhance genomic breeding for several crops, such as tomato [93] and grape [94]. Furthermore, it is essential to assemble new high-quality genomes using long-read sequencing technologies such as PacBio and Oxford Nanopore.

Second, a user-friendly *Phal.*-specific database should be established to systematically document detailed horticultural traits. Such a database would serve as a valuable resource for GWAS by allowing users to access, download, and upload morphological data. In addition, via the database, breeders can visualize the existing phenotypic landscape, and identify untapped trait combinations for future breeding. For example, while cultivars with large, sub-rotund petals typically exhibit white, yellow, pink, or purple-red colors, those with rare colors—such as purple-blue, true red (called ‘Chinese red’), brown, or rainbow—often have smaller, lanceolate, or ovate petals (Fig. 4A). It would be of great ornamental and economic value to breed novel cultivars with both large, sub-rotund petals and those scarce colors.

Third, genotype-independent transformation technologies [95, 96] could help overcome the challenges in establishing a stable transformation system for *Phal.* orchids. These methods enable direct genetic modification via protocorm-like bodies, bypassing trait segregation issues commonly associated with protocorms—the conventional explants used in transgenic experiments involving hybridization.

In summary, further effects are required to comprehensively document the diversity of floral traits in *Phal.* orchids, decipher their evolutionary history and genetic basis, and improve the reference genome quality and transgenic technology systems to support future genomics-assisted and molecular breeding for novel *Phal.* cultivars.
